# Structured lifestyle modification as an adjunct to obesity pharmacotherapy: there is much to learn

**DOI:** 10.1038/s41366-024-01499-2

**Published:** 2024-03-08

**Authors:** Enda Murphy, Francis Martin Finucane

**Affiliations:** 1 Department of Medicine, College of Medicine, Nursing and Health Sciences, University of Galway, Galway, Ireland; 2 Bariatric Medicine Service, Centre for Diabetes, Endocrinology and Metabolism, Galway University Hospitals, Galway, Ireland; 3 Cúram, University of Galway, Galway, Ireland

**Keywords:** Obesity, Obesity

## Abstract

We are at the start of an exciting new era of very effective pharmacotherapy for patients with obesity, with the latest generation of drugs approaching the efficacy of obesity surgery. Clinical trials of obesity drugs tend to emphasise the importance of participation in some form of structured lifestyle intervention for all trial participants. This usually consists of advice to reduce calorie intake and increase moderate to vigorous physical activity. There is strong evidence that structured lifestyle modification programmes improve health in patients with obesity and related disorders. However, there is no specific evidence that they improve the response to obesity medications. This is because of the way that drug trials for patients with obesity have traditionally been designed, with participants in the active drug treatment group being compared to participants on placebo drug treatment, but with both groups always receiving the same structured lifestyle intervention. While this approach is entirely reasonable, it makes it impossible to draw any inferences about the efficacy of structured lifestyle modification to augment the response to drug therapy. Given this genuine equipoise, a critical step in ensuring that our treatment of patients with obesity is robustly evidence-based is to determine whether “drug plus lifestyle” offer any advantage over “drug plus placebo” in large, well-designed and adequately powered clinical trials. We also need to determine the cost-effectiveness of these programmes.

## Introduction

The latest generation of obesity medications has broken new ground, surpassing the benchmark of 10% weight loss in most of the patients enroled in clinical trials [1]. Semaglutide [2], a glucagon-like peptide-1 receptor agonist (GLP-1RA), is the first of these new agents to obtain regulatory approval for the treatment of obesity, with tirzepatide [3] and other drugs [4] showing even stronger potential. Lifestyle modification is the cornerstone of the therapeutic approach to obesity and is universally incorporated into clinical trials of new obesity drugs. However, uncertainties exist about the role that structured lifestyle modification programmes should play for patients who are starting drug therapy for treatment of their obesity. Of course, optimising dietary intake and physical activity levels in any patient with obesity is desirable and sensible, but whether a pre-defined structured intervention of fixed duration, modality, frequency or intensity improves the efficacy of drug treatment is unclear. We sought to identify areas of uncertainty in the evidence base for lifestyle intervention in patients starting drug therapy for treatment of obesity and to propose some scientific questions that could be prioritised by clinical researchers and healthcare system managers seeking to optimise effective and efficient care for people with obesity.

## Evidence for structured lifestyle modification in patients with obesity

Several well-conducted randomised controlled trials have shown the benefits of structured lifestyle modification programme patients with cardiovascular disease [5], impaired glucose metabolism [6] and type 2 diabetes [7, 8]. For example, the Look AHEAD [9] (Action for Health in Diabetes) trial recruited over 5000 participants with type 2 diabetes and an average body mass index of 36 kgm^-2^ who were randomised to “usual care” diabetes structured education or to “intensive lifestyle intervention”, with an ambitious weight loss target of 10%, using meal replacement, if necessary, to achieve that goal. After ten years of follow-up, the intensive lifestyle intervention group lost more weight and had improvements in many important health related outcomes such as diabetes control and medication usage [10], but with no difference in the primary outcome of major adverse cardiovascular events. This may have been due to a lower incidence than expected of cardiovascular events in the control group, and the overwhelming health improvements from intensive lifestyle intervention demonstrated in the trial should not be dismissed.

Drop-out from intensive lifestyle modification programmes can be very high in “real world” clinical studies [11, 12], total weight loss is often modest [13] and can be difficult to maintain in the longer term [14, 15]. While 10% weight loss is generally regarded as a meaningful level with which to improve health [8, 16], it is rarely achieved with lifestyle modification alone, in stark contrast to the new generation of obesity drugs. In our own hospital-based regional referral centre for patients with severe obesity, only 12.8% of patients completing a ten-week, group-based, multidisciplinary structured diet and physical activity programme had reductions in body weight of 5% or more [13, 17].

Structured lifestyle modification programmes are rarely offered in isolation in specialised multidisciplinary obesity clinics and are usually combined with surgical or pharmacological interventions. However, evidence that pre-operative lifestyle modification programmes are beneficial in patients undergoing bariatric surgery is also relatively weak, with methodological inconsistencies in some studies [18] and very few high-quality randomised controlled trials [19] being well-described limitations [20]. In well-conducted clinical trials, results have not shown any benefits from structured lifestyle modification prior to bariatric surgery. In one trial, the only difference in participants who completed a structured lifestyle intervention before bariatric surgery was that, somewhat unexpectedly, they lost less rather than more weight in the years after surgery [21]. Despite the lack of evidence, some jurisdictions and healthcare organisations still require patients to undertake mandatory structured lifestyle modification programmes before proceeding to surgery, which can act as a barrier to care [22] and is contrary to European [23] and American [24] clinical practice guidelines.

## Lifestyle modification in obesity drug trials

A noteworthy feature of all modern phase three drug trials of obesity is the inclusion of some form of lifestyle modification strategy as a requirement for patients in the active treatment and placebo arms, as summarised in Table 1. This typically consists of advice to consume 500 kcal less per day than the participant’s estimated calorie requirements and to participate in moderate to vigorous intensity physical activity for at least 150 min per week. This advice is generic and is delivered to individuals either by a dietitian or by another suitably qualified healthcare professional. Of note, trials of the same drugs for treating type 2 diabetes rather than obesity tend to have a much lower emphasis on the importance of lifestyle behaviours in their design. For example, the LEAD (Liraglutide Effect and Action in Diabetes) suite of six clinical trials sought to evaluate the effectiveness of liraglutide on diabetes control when added to various other diabetes medications, but none of the papers describing the treatment strategies in these trials mentions structured lifestyle modification, calorie restriction (or any other dietary strategy) or the setting of physical activity targets for participants to meet [25–30]. In contrast, the SCALE (Satiety and Clinical Adiposity- Liraglutide Evidence) trials, which sought to determine the effectiveness of a higher dose of liraglutide for the treatment of obesity rather than diabetes, emphasised the need for all participants to receive lifestyle counselling at intervals throughout the respective studies, which tended to focus on a target daily calorie deficit of 500 kcal and a weekly physical activity target of 150 min [31]. The LEADER trial (Liraglutide Effect and Action in Diabetes: Evaluation of Cardiovascular Outcome Results) [32], which demonstrated a reduction in cardiovascular events in patients with type 2 diabetes treated with liraglutide noted that “…at the time of randomisation the importance of lifestyle approaches for diabetes management including dietary changes, physical activity and weight management should be stressed to all subjects”, but there was no stipulation for supervised setting of individual participants’ dietary and physical activity goals.

**Table 1:** Changes in body weight in active drug treatment versus placebo (lifestyle modification alone) groups in selected recent obesity drug trials.

Trial Name	Author/ year (reference)	Medication and Dose	Trial Duration (Weeks)	Lifestyle Intervention Features	Number of Participants	Weight Loss with Active Drug Treatment (%)	Weight Loss with Lifestyle Intervention Alone (%)
LEADER	Marso/ 2013 [38]	Liraglutide 1.8 mg	60	The importance of lifestyle approaches for diabetes management including dietary changes, physical activity and weight management was stressed to all participants.	9340	5.8%	3.3%
SCALE	Pi-Sunyer/ 2015 [31]	Liraglutide 3.0 mg	56	Dietary intervention seeking a 500 kcal/ day deficit, as well as a target of 150 minutes of physical activity per week.	3731	8%	2.6%
MODEL	Wadden/ 2018 [70]	Liraglutide 3.0 mg	52	Dietary intervention seeking a 500 kcal/ day deficit, as well as a target of 150 minutes of physical activity per week. A second arm received a 1200 kcal, 12-week meal replacement diet programme.	150	11.5%	6.1%
STEP1	Wilding/ 2021 [2]	Semaglutide 2.4 mg	68	Dietary intervention seeking a 500 kcal/ day deficit, as well as a target of 150 minutes of physical activity per week.	1961	14.9%	2.4%
STEP 2	Davies/ 2021 [43]	Semaglutide 1 and 2.4 mg	68	Dietary intervention seeking a 500 kcal/ day deficit, as well as a target of 150 minutes of physical activity per week.	1210	6.9 and 9.6%	3.4%
STEP 3	Wadden/ 2021 [46]	Semaglutide 2.4 mg	68	Dietary intervention seeking a 500 kcal/ day deficit, as well as a target of 150 minutes of physical activity per week. A second arm received a 1000-1200 kcal, 8-week meal replacement diet programme, followed by a hypocaloric (1200-1800 kcal) diet for 68 weeks, and with 100 minutes of physical activity per week increasing to 200 minutes per week gradually by 16 weeks.	567	16%	5.7%
STEP 4	Rubino/ 2022 [48]	Semaglutide 2.4 mg	75	Dietary intervention seeking a 500 kcal/ day deficit, as well as a target of 150 minutes of physical activity per week.	902	18%	5%
STEP 5	Garvey/ 2021 [45]	Semaglutide 2.4 mg	68	Dietary intervention seeking a 500 kcal/ day deficit, as well as a target of 150 minutes of physical activity per week.	304	15.2%	2.6%
STEP 6	Kadowaki/ 2022 [47]	Semaglutide 1.7 and 2.4 mg	68	Dietary intervention seeking a 500 kcal/ day deficit, as well as a target of 150 minutes of physical activity per week.	401	9.6 and 13.2%	2.1%
STEP 8	Rubino/ 2022 [48]	Semaglutide 2.4 mg	68	Dietary intervention seeking a 500 kcal/ day deficit, as well as a target of 150 minutes of physical activity per week.	338	15.8%	6.4%
SURMOUNT 1	Jastreboff/ 2022 [3]	Tirzepitide 5, 10 and 15 mg	72	Dietary intervention seeking a 500 kcal/ day deficit, as well as a target of 150 minutes of physical activity per week.	2539	15, 19.5 and 20.9%	3.1%
SURMOUNT 2	Garvey/ 2023 [71]	Tirzepitide 10 and 15 mg	72	Dietary intervention seeking a 500 kcal/ day deficit, as well as a target of 150 minutes of physical activity per week.	1514	12.8 and 14.7%	3.2%
Retatrutide	Rosenstock/ 2023 [54]	Retatrutide 12 mg	36	None (Phase 2 trial)	281	16.9%	2%
Retatrutide	Jastreboff/ 2023 [4]	Retatrutide 12 mg	48	None (Phase 2 trial)	338	24.2%	2.1%

Similarly, in the SUSTAIN suite of clinical trials describing the influence of semaglutide on glycaemic and cardiovascular outcomes in patients with type 2 diabetes [33–42], investigators noted that “lifestyle modifications and metformin are considered foundational therapy in most countries”, but did not describe any sort of specific lifestyle intervention for trial participants. However, when semaglutide was studied as a treatment for obesity during the STEP (Semaglutide Treatment Effect in People with obesity) trials [2, 43–48], the focus again was on a target daily calorie deficit of 500 kcal and a weekly physical activity target of 150 minutes, provided by a dietician or other qualified health care professional every four weeks, either in person or by telephone. Moreover, participants were encouraged to keep food and activity diaries in either paper form or using a tracker app, for review and discussion at each consultation.

More recently, trials of tirzepatide for treatment of type 2 diabetes in the SURPASS trials [49–53] did not stipulate any form of structured lifestyle modification, whereas in the SURMOUNT trial (of the same drug, but for the treatment of obesity) [3], participants in both the active treatment and control groups underwent regular counselling sessions delivered by a healthcare professional aimed at adherence to a balanced diet and a daily calorie deficit of 500 kcal, with at least 150 minutes of physical activity per week. The latest obesity drug to be evaluated in clinical trials, retatrutide (a GLP-1, GIP and glucagon triple receptor agonist), has only recently undergone phase-two studies for type 2 diabetes [54] and obesity [4], without a lifestyle intervention component in either study. The current approach to clinical trial design would suggest that the manufactures of these new and very promising drugs for type 2 diabetes and obesity view lifestyle modification as an important adjunct to pharmacotherapy for the latter but not so much for the former.

What does this apparently inconsistent view of two equally “lifestyle-related” diseases say about our approach to obesity treatment, and what implications might it have for future clinical research and practice? Firstly, the disparity between our approach to obesity and diabetes when it comes to utilising these promising new drugs is real, as epitomised by the recent critical global shortages of semaglutide. These shortages have been driven by unprecedented awareness of (and demand for) semaglutide and have had knock-on effects on patients who have run out of the drug or have not been able to start it. The scarcity has highlighted a culture of institutionalised bias and discrimination against people with obesity within the medical establishment and the pharmaceutical industry [55]. In several jurisdictions, including Ireland, the UK, US and Australia, healthcare regulatory authorities have urged prescribers to use semaglutide only for patients with type 2 diabetes, to preserve this scarce resource for those perceived to need it most. This implies that obesity is inherently less worthy of treatment than diabetes, contrary to the recognition of obesity as a disease [56] and trivialising the proven clinical benefits of the treatment of obesity [2, 3, 57]. The misgivings about the appropriateness of doctors legitimately prescribing safe, effective medications (within their licenced indication, if at a lower dose than stipulated in their marketing authorisation) for patients with obesity are entirely consistent with the apparent belief that patients with obesity need to try harder to modify their lifestyle than patients with type 2 diabetes.

Often, access to publicly funded lifestyle modification programmes for patients is very limited, which could further constrain access to obesity drug treatment. While individualised lifestyle assessment and education in the obesity clinic is important, insisting that patients undertake structured lifestyle modification while on drug therapy, in the absence of any evidence of benefit, could reinforce the view of many healthcare professionals including doctors [58] and nurses [59], that obesity arises from poor discipline, motivation or effort. It could lead to patients avoiding care, compounding low esteem, self-stigmatisation [60], with worse subsequent outcomes [61].

## What are the scientific priorities in this field?

Our current clinical practice is to offer patients with obesity attending our service who are considering drug therapy for obesity an opportunity to participate in ten-week supervised, group-based structured lifestyle modification programme with nursing, dietetic and physical activity expertise [13]. They can do this before, during or after initiating drug therapy, or not at all - it is not compulsory, especially when the patient has previously completed a structured programme elsewhere. The focus of our multidisciplinary team is not to try to motivate the patient to lead a more responsible lifestyle, rather it is to empower them and ensure the nutritional adequacy of their diet. However, this clinical approach is not robustly evidence based. In fact, no study has ever determined whether participation in a structured lifestyle modification such as described in the trials in Table 1 does anything to improve the response to drug treatment. The current approach to clinical trial design for obesity drugs needs to be turned on its head, so that formal, rigorous and meaningful comparisons can be made between “drug and lifestyle” and “drug and placebo” groups. These studies should include robust health economic and cost effectiveness analyses to determine if, how and at what cost the addition of structured lifestyle modification to drug therapy is beneficial. It may be that group-based lifestyle interventions are more effective than individual ones in patients on drug therapy for obesity, consistent with findings from other lifestyle intervention studies [62]. This would have important implications for costs and configuration of current clinical pathways for obesity.

Future trials of lifestyle interventions to augment the response to obesity drugs should probably include some form of physical activity component. We have noted that patients attending our group-based structured lifestyle programme lost more weight if they were physically active [63]. This is consistent with findings in patients who had bariatric surgery [64] and in the Look AHEAD trial [65, 66], where increased physical activity was shown to be associated with greater longer term weight loss. Current clinical practice guidelines recommend combining dietary and physical activity components in structured lifestyle programmes [67, 68], which should inform the design of lifestyle interventions for trials to augment drug therapy response.

Such trials could also help to address some of the controversy around the specific types of dietary strategies advocated in lifestyle modification programmes. For example the widely used dietary strategy of asking patients to reduce their intake by 500 kcal below their calculated requirements per day [69] seems to miss the point that this is easier said than done. Ultimately, whether a dietary intervention involves alterations in macronutrient composition, time restricted eating or intermittent fasting, the best one will be that which the patient can adhere to and afford. Also, whether ultra-processed foods should be excluded, or carbohydrates restricted is very unclear, but warrants consideration in future trials. A further area of uncertainty that needs to be addressed in future studies is the impact of specialist dietetic input on malnutrition-related outcomes (such as micronutrient deficiencies) associated with reduced oral intake in patients taking drugs for obesity.

Until large, adequately powered multicentre randomised controlled trials identify which structured lifestyle modification programmes help patients to respond better to drugs, they should not be a compulsory component of treatment. Multidisciplinary dietetic and physical activity expertise is essential in the care of patients with obesity, but it should be used to empower and educate them. The widely adopted but potentially over-simplistic approach of advising drug trial participants to consume 500 fewer calories per day and be more active requires robust evaluation of its efficacy and cost-effectiveness. It may be that dietetic and multidisciplinary expertise could be better deployed in more nuanced and individualised ways.
